# Prevalence and correlates of domain-specific sedentary time of adults in the Netherlands: findings from the 2006 Dutch time use survey

**DOI:** 10.1186/s12889-019-6764-7

**Published:** 2019-06-03

**Authors:** Anne Loyen, Josephine Y. Chau, Judith G. M. Jelsma, Femke van Nassau, Hidde P. van der Ploeg

**Affiliations:** 1 Department of Public and Occupational Health, Amsterdam Public Health research institute, VU University Medical Center, Van der Boechorststraat 7, 1081 Amsterdam, BT the Netherlands; 2 Prevention Research Collaboration, Sydney School of Public Health and Charles Perkins Centre, the University of Sydney, Sydney, 2006 NSW Australia; 3 Centre for Nutrition, Prevention and Health Services, National Institute for Public Health and the Environment, Antonie van Leeuwenhoeklaan 9, 3721 Bilthoven, MA the Netherlands

**Keywords:** Adults, Correlates, Epidemiology, Netherlands, Prevalence, Sedentary behaviour, Time use

## Abstract

**Background:**

Relatively little is known about how total sedentary time is accumulated in different domains and if correlates of sedentary time differ across domains. Time use surveys present a unique opportunity to study sedentary time in more detail. This study aimed to use the 2006 Dutch time use survey to 1) describe the (sedentary) time use of Dutch adults, and 2) explore socio-demographic and health-related correlates of total (non-occupational) and domain-specific sedentary time.

**Methods:**

The Dutch time use survey randomly selected participants from a population-representative research sample of Dutch households. Participants reported daily activities on seven consecutive days using a time use diary and socio-demographic and health-related characteristics during telephone interviews. All reported activities were coded for activity domain (i.e. education; household; leisure; occupation; sleep; transport; voluntary work) and activity intensity (i.e. sedentary; light intensity physical activity; moderate-to-vigorous intensity physical activity). As occupational activities were not specified in sufficient detail, the intensity of these activities was unknown. We described the time spent in different domains and intensities, and assessed the socio-demographic and health-related correlates of high levels of total (non-occupational), household, leisure, and transport sedentary time using logistic regression analyses.

**Results:**

The final dataset consisted of 1614 adult (18+) participants. On average, participants spent 8.0 h (61.1%) of their daily waking non-occupational time on sedentary activities. More than 87% of leisure time was spent sedentary. Men, participants aged 18–34 and 65+ years, full-time employed participants and obese participants had higher levels of total non-occupational sedentary time. The correlates of household, leisure and transport sedentary time differed by domain.

**Conclusions:**

This study reports high levels of total non-occupational sitting time of Dutch adults. The large proportion of sedentary leisure activities might indicate the potential of strategies aiming to reduce leisure sedentary time. The difference in correlates across sedentary behaviour domains demonstrates the importance of targeting these domains differently in interventions and policies.

## Background

Too much sitting, or sedentary behaviour, is becoming a well-known health risk [1]. Sedentary behaviour is characterised by a low energy expenditure whilst in a sitting, reclining, or lying position [1]. Sedentary behaviours can be classified by the activity domains in which they take place, such as occupation (e.g. working at a desk), leisure (e.g. watching television), and transport (e.g. driving a car). High levels of sedentary behaviour are associated with increased risk of type 2 diabetes, cardiovascular disease, some cancers, and mortality [2, 3]. The risk of all-cause mortality is shown to increase if adults accumulate more than 6 to 8 h of sedentary time per day [3, 4].

Current estimates suggest that a large proportion of the adult population accumulates high levels of sedentary time [5, 6]. According to a European survey, for example, over 18% of European adults reported to sit more than 7.5 h/day, ranging from 9% in Spain to 32% in the Netherlands [6]. The majority of surveillance studies to date used questionnaires to assess sedentary behaviour [7, 8]. While questionnaires are most feasible to use in large-scale surveys, they are prone to measurement issues such as recall bias and social-desirability bias and often underestimate actual sedentary time [9, 10]. Moreover, questionnaires often ask about the total time spent sitting on a usual day, without assessing the specific types of sedentary behaviour or the domain in which the behaviours occurred [7, 8], limiting the understanding of the nature and context of the sedentary behaviours and thus the potential for translation to domain-specific interventions and policies aiming to reduce sedentary time.

In the last decade, time use surveys have been recognised as a methodology that overcomes (most of) these issues with questionnaires, as they collect detailed information on daily activities of the current day without an obvious focus on health-related behaviours. In these surveys, participants complete a diary in which they report all daily activities in a certain time interval (often every 5 or 10 min) for a certain number of days (mostly 2–7 days). This provides very detailed information about participants’ specific behaviours. Time use surveys have been proven to be a valid method to monitor non-occupational sedentary behaviour [11]. In the Netherlands, time use surveys have been conducted every five years since 1975. A trend analysis of the surveys between 1975 and 2005 showed substantial changes in non-occupational sedentary behaviour, with large increases in sedentary transport time and screen time [12].

In 2006, the methodology of the Dutch time use survey was adapted to match the Harmonized European Time Use Survey (HETUS) [13], which was shown to provide more detailed and more valid data than the previous method and allowed for better comparability between countries [14]. The 2006 survey also collected a range of socio-demographic and health-related characteristics of the participants. The aim of the current study was therefore to use the 2006 Dutch time use survey to 1) describe the (sedentary) time use of Dutch adults, and 2) explore socio-demographic and health-related correlates of high levels of total (non-occupational) and domain-specific sedentary time.

## Methods

This study used data from the 2006 Dutch time use survey, which was a government-initiated survey commissioned by the Netherlands Institute for Social Research, and conducted as part of the Mobility Research Netherlands [15]. For that research, 34,603 Dutch households were randomly selected based on postal address, and within each household a maximum of five residents could respond, resulting in a total of 53,545 participants (≥10 years old). A random, population-representative selection of 3041 of the Mobility Research Netherlands participants were invited to additionally participate in the time use survey. The time use survey was conducted throughout 2006 and consisted of different components, which are described in detail in the (Dutch) fieldwork report [16]. In short, potential participants were invited by telephone by trained interviewers (multiple attempts). Participants who were willing to participate received written information and provided verbal informed consent by telephone. Subsequently, participants reported several socio-demographic characteristics during a telephone interview and were asked to complete the time use diary that was sent to them by mail. During the completion period participants were contacted by telephone twice, to discuss the previous diary day in detail to ensure high data quality. All participants were contacted at their second diary day to discuss the first day of their diary. The second phone call was planned together with the participant and thus differs between participants. After completing the diary, participants were interviewed by telephone once more about a range of topics, including health-related characteristics.

The time use diary consisted of seven days, each starting and ending at 04:00 a.m. Participants were asked to write down what they were doing (primary and secondary activity), where, and with whom in 10-min intervals on a daily basis. Participants completed their diary in their own words, which were later coded by trained coders using the HETUS activity codes [17]. Subsequently, all primary activities were assigned to only one of the following activity domains: education, household, leisure, occupation, sleep, transport and voluntary work. In addition, all primary activities were also assigned one activity intensity, based on the metabolic equivalent (MET) score: sedentary (≤1.5 MET), light intensity physical activity (LPA; 1.6–2.9 MET), and moderate-to-vigorous intensity physical activity (MVPA; ≥3 MET). These classifications were derived from the Compendium of Physical Activities [18] and the coding system used by the American Time Use Survey [19]. The MET-classification has been validated against accelerometers [11], and was used in previous physical activity and sedentary behaviour research [12, 20]. As occupational activities were not specified in sufficient detail, the activity intensity of these activities could not be classified using MET-scores and were therefore not included in analyses.

The socio-demographic and health-related characteristics consisted of gender, age, educational level, work status, self-reported health, mobility issues, weight status, sleep time, smoking behaviour, drinking alcohol, eating take-away food, and happiness. Age was categorised into 18–34; 35–49; 50–64; 65+ years old. Educational level was based on the highest attained education and categorised into low (primary and pre-vocational secondary education); middle (secondary education); high (higher professional and university education). Work status was categorised into not working; working part-time; working full-time. The difference between part-time and full-time was self-perceived as there was no predefined cut-point (e.g. 32 or 36 h/week). Self-reported health was categorised into very good/good; not good (including: ‘okay’, ‘sometimes bad - sometimes good’, ‘bad’). Mobility was assessed by asking whether the participant had difficulty walking, taking the stairs, or moving outdoors and was dichotomised into no issues; issues (including: some difficulty, a lot of difficulty, impossible). Self-reported height and weight were used to calculate Body Mass Index (BMI) and categorised into normal weight (BMI 18.5–24.9 kg/m^2^); overweight (BMI 25.0–29.9 kg/m^2^); obese (BMI ≥30.0 kg/m^2^) [21]. Average sleep time was based on the time use diary data and dichotomised into healthy (7–9 h/day); unhealthy (< 7 h/day or > 9 h/day) [22]. Participants were classified as non-smokers; smokers based on their current smoking behaviour. Drinking alcohol was categorised into never; 1–7 glasses/week; > 7 glasses/week. Eating take-away food was categorised into never; less than once a month; once a month or more. Happiness was dichotomised into very happy/happy; not happy (including: ‘not happy - not unhappy’, ‘not very happy’, ‘unhappy’).

The data was accessed through the Dutch Data Archiving and Networked Services [23]. All analyses were performed in SPSS, version 22. For each participant, daily summary values were calculated across the seven diary days – using the activities reported in 10-min intervals. In the current analyses, only the activities listed as primary activities were taken into account, ignoring possible secondary activities (such as listening to the radio (secondary) while performing household chores (primary)). The time spent in different activity domains and activity intensities were explored using descriptive analyses. The levels of sedentary time were calculated as absolute minutes/day, and as the proportion of the waking non-occupational time/day - to account for the varying amounts of sleep time and occupational time per participant. The socio-demographic and health-related correlates of high levels of total (non-occupational), household, leisure, and transport sedentary time were assessed using logistic regression analyses. As meaningful cut-points for the sedentary domains are currently lacking, “high levels” were defined as ≥75th percentile of the proportion of waking non-occupational time, calculated separately for each sedentary domain. Univariate models were used to identify statistically significant correlates (*p* < 0.05), after which the significant correlates were combined in multivariable models.

In order to still gain some insight into (the influence of) occupational sedentary time for which activity intensities are not available in the current study, the time spent in non-occupational activity intensities was explored in full-time employed participants only. Full-time employed participants were classified using a question separate to the time use diary to distinguish between participants who reported to never use a computer at work, use a computer ≤4 h/workday, and > 4 h/workday. Using the “computer use at work” question as a proxy indication for total occupational sedentary time, these analyses were conducted to compare daily activity patterns across participants with different levels of occupational computer time.

## Results

Of the 3041 participants that were randomly selected, 2811 participants (92%) were successfully contacted, 2190 (72%) were willing to participate, and 1875 (62%) provided complete data. A comparison with the general Dutch population showed that the research sample was similar in terms of gender distribution, but that people aged 18–29 years were underrepresented in the time use survey [16]. We excluded 240 participants who were younger than 18 years, and 21 underweight participants (BMI < 18.5 kg/m^2^), leaving 1614 participants in the final dataset. Of these participants, 53% were women, and the mean age (SD) was 49 (16) years (ranging from 18 to 93 years old). All socio-demographic and health-related characteristics of the participants are presented in Table 1.

**Table 1:** Socio-demographic and health-related characteristics of the study population

N (%) All participants	1614 (100%)
Gender	
N (%) Male	755 (46.8%)
N (%) Female	859 (53.2%)
Age	
Mean (SD) age	49.0 (16.1)
N (%) 18–34 years old	319 (19.8%)
N (%) 35–49 years old	539 (33.4%)
N (%) 50–64 years old	441 (27.3%)
N (%) ≥65 years old	315 (19.5%)
Educational level	
N (%) Low	521 (32.3%)
N (%) Middle	568 (35.2%)
N (%) High	525 (32.5%)
Work status	
N (%) Not working	603 (37.4%)
N (%) Part-time	439 (27.2%)
N (%) Full-time	571 (35.4%)
Self-reported health	
N (%) Very good/good	1379 (85.7%)
N (%) Not good	231 (14.3%)
Mobility issues	
N (%) No issues	1410 (87.5%)
N (%) Issues	201 (12.5%)
Weight status	
Mean (SD) BMI	25.0 (3.7)
N (%) Normal (BMI 18.5–24.9)	855 (55.3%)
N (%) Overweight (BMI 25.0–29.9)	546 (35.3%)
N (%) Obese (BMI ≥30.0)	144 (9.3%)
Sleep time	
Mean (SD) hours/day sleep time	8.6 (1.1)
N (%) Sleeping 7–9 h/day	1043 (64.6%)
N (%) Sleeping < 7 or > 9 h/day	571 (35.4%)
Smoking behaviour	
N (%) No	1257 (77.9%)
N (%) Yes	356 (22.1%)
Drinking alcohol	
N (%) Never	398 (26.0%)
N (%) 1–7 glasses/week	715 (46.7%)
N (%) > 7 glasses/week	417 (27.3%)
Eating take-away food	
N (%) Never	429 (26.6%)
N (%) Less than once a month	478 (29.7%)
N (%) Once a month or more	704 (43.7%)
Happiness	
N (%) Very happy/happy	1518 (94.4%)
N (%) Not happy	90 (5.6%)

*N* number of participants*, SD* Standard Deviation*, BMI* Body Mass Index

**Fig. 1:**
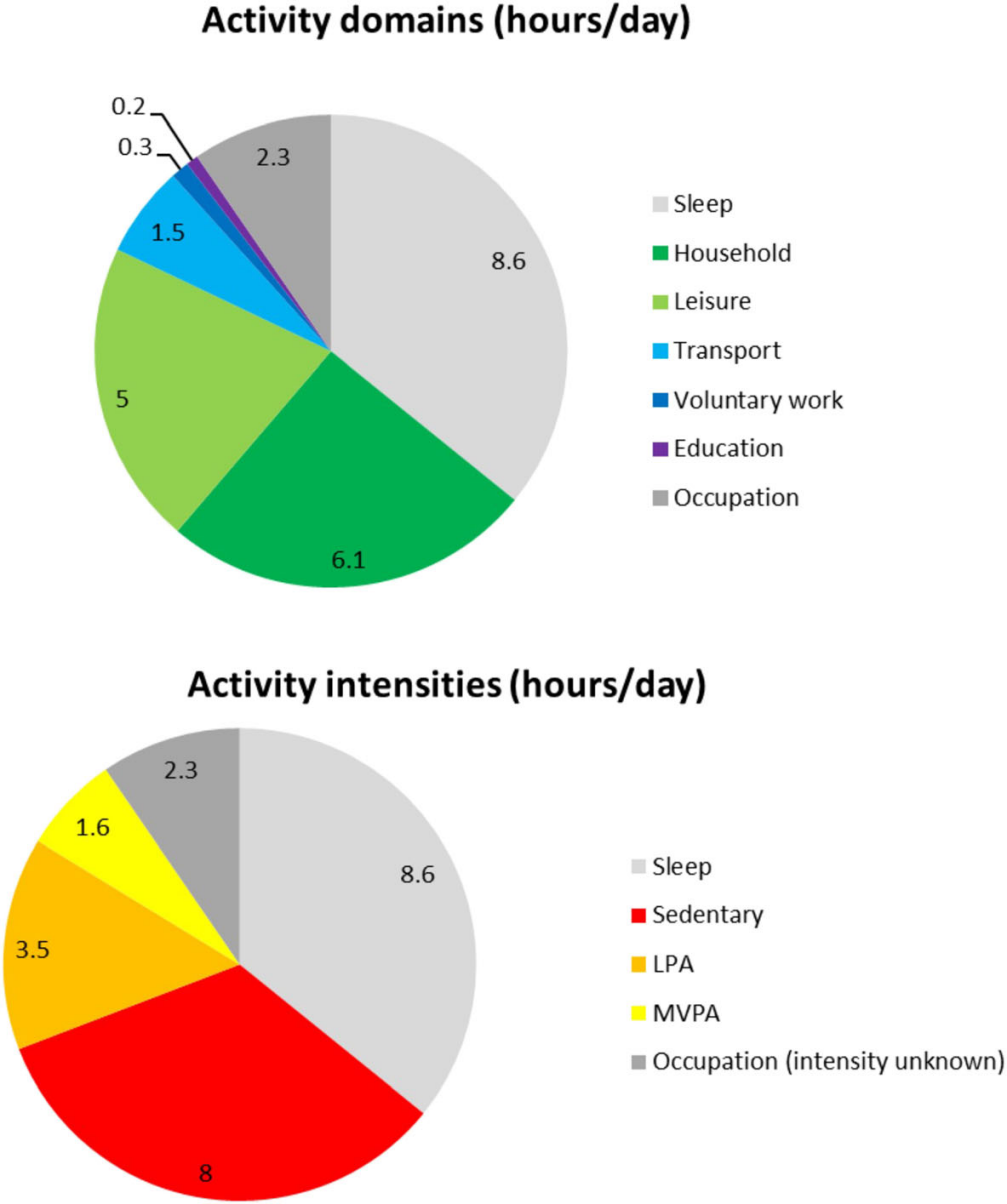
The average time (hours/day) spent in different activity domains (above) and activity intensities (below). As occupational activities were not further specified, the intensity of these activities is unknown. *LPA = light intensity physical activity; MVPA = moderate to vigorous intensity physical activity*

The average time/day participants spent in the different activity domains and activity intensities is shown in Fig. 1. In terms of activity domains, most time was spent on sleep, household activities and leisure activities. In addition, participants spent 8.0 h of their waking, non-occupational time/day on sedentary activities, 3.5 h/day on LPA and 1.6 h/day on MVPA.

**Fig. 2:**
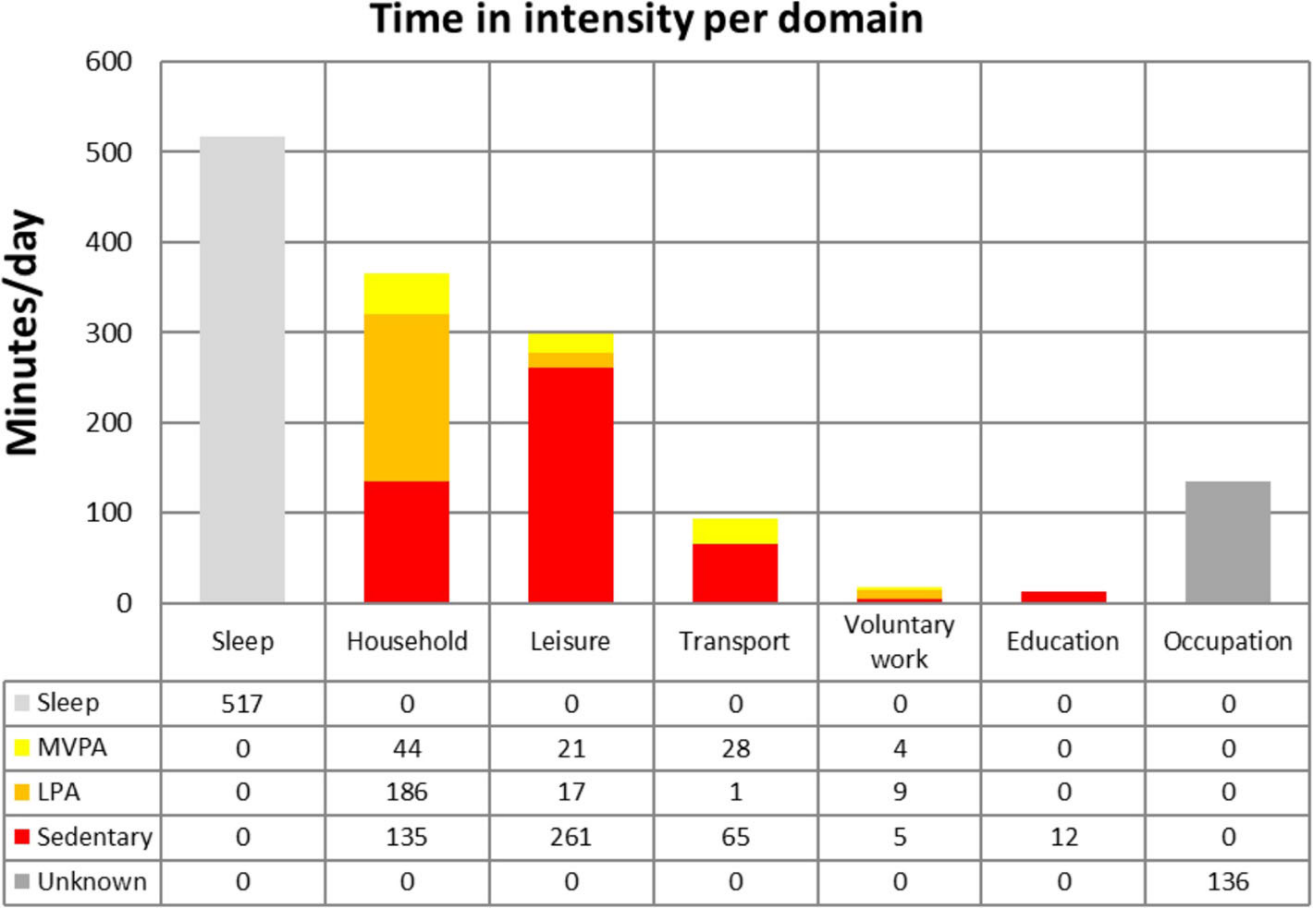
The average time (minutes/day) spent in different activity intensities in each activity domain. As occupational activities were not further specified, the intensity of these activities is unknown. *LPA = light intensity physical activity; MVPA = moderate to vigorous intensity physical activity*

Figure 2 shows the average time participants spent in different activity intensities in each activity domain. In the household domain, half of the time was spent on LPA. The majority of the leisure (87%) and transport time (70%) was spent sedentary. Voluntary work was a mix of different intensities and all time spent on education was sedentary.

The levels of total, household, leisure and transport sedentary time are summarised in Table 2. Overall, Dutch adults were sedentary for 8.0 h (61%) of the daily waking non-occupational time. Of that time, 17% was spent on household activities, 33% on leisure pursuits, and 9% on transport.

**Table 2:** Levels of total (non-occupational), household, leisure and transport sedentary time

	Total sedentary time		Household sedentary time		Leisure sedentary time		Transport sedentary time	
	Minutes/day	Percentage	Minutes/day	Percentage	Minutes/day	Percentage	Minutes/day	Percentage
Overall	478.2 (107.2)	61.1%	135.4 (49.0)	17.4%	261.0 (107.2)	33.0%	64.7 (44.5)	8.6%
Gender								
Male	496.0 (116.9)	65.9%	129.8 (46.5)	17.6%	276.0 (120.6)	36.0%	73.2 (50.5)	10.1%
Female	462.6 (95.3)	56.8%	140.3 (50.7)	17.2%	247.9 (91.9)	30.3%	57.2 (36.9)	7.2%
Age								
18–34 years old	451.5 (109.8)	62.1%	112.3 (43.3)	15.5%	223.0 (81.4)	30.9%	70.3 (39.7)	10.0%
35–49 years old	432.6 (82.0)	58.4%	135.7 (47.1)	18.2%	214.9 (78.2)	28.9%	70.8 (45.2)	9.8%
50–64 years old	493.7 (94.6)	62.1%	141.4 (47.3)	17.9%	274.8 (98.9)	34.3%	66.8 (46.5)	8.6%
≥ 65 years old	561.5 (105.8)	63.1%	150.1 (52.0)	17.0%	359.2 (114.1)	40.3%	45.6 (39.8)	5.1%
Educational level								
Low	498.2 (107.8)	60.3%	151.0 (55.0)	18.4%	286.2 (112.4)	34.3%	52.8 (40.3)	6.6%
Middle	463.7 (105.3)	60.6%	130.0 (42.8)	17.0%	246.6 (99.4)	32.0%	69.5 (44.9)	9.3%
High	474.0 (105.9)	62.4%	125.9 (45.2)	16.7%	251.7 (105.7)	32.8%	71.3 (45.7)	9.8%
Work status								
Not working	538.6 (103.6)	60.5%	149.1 (52.6)	16.8%	324.7 (112.9)	36.4%	50.1 (38.8)	5.7%
Part-time	446.2 (92.7)	56.9%	132.3 (43.4)	16.9%	224.1 (78.2)	28.6%	65.6 (41.4)	8.4%
Full-time	439.0 (91.6)	64.9%	123.3 (45.6)	18.3%	222.2 (86.5)	32.7%	79.5 (47.4)	11.8%
Self-reported health								
Very good/good	473.4 (105.1)	61.0%	133.9 (48.4)	17.3%	254.1 (103.6)	32.4%	66.9 (45.0)	9.0%
Not good	506.7 (116.0)	61.3%	144.4 (52.1)	17.6%	301.6 (118.4)	36.2%	51.7 (38.8)	6.4%
Mobility issues								
No issues	471.7 (104.7)	61.0%	133.6 (47.4)	17.3%	253.3 (103.1)	32.4%	66.8 (44.5)	9.0%
Issues	522.2 (113.7)	61.1%	148.5 (57.7)	17.5%	316.1 (119.1)	36.7%	49.7 (41.2)	5.9%
Weight status								
Normal (BMI 18.5–24.9)	461.6 (103.6)	60.0%	132.4 (48.1)	17.3%	244.3 (98.8)	31.5%	63.9 (41.9)	8.6%
Overweight (BMI 25.0–29.9)	489.2 (105.1)	62.0%	137.8 (48.3)	17.5%	273.2 (108.0)	34.2%	67.0 (48.2)	8.8%
Obese (BMI ≥30.0)	517.5 (115.1)	63.4%	138.7 (53.1)	17.0%	302.8 (126.0)	36.6%	64.2 (45.3)	8.3%
Sleep time								
Sleeping 7–9 h/day	479.9 (108.3)	60.9%	135.3 (47.7)	17.2%	259.5 (108.8)	32.5%	68.3 (45.7)	9.0%
Sleeping < 7 or > 9 h/day	475.0 (105.2)	61.4%	135.6 (51.3)	17.6%	263.8 (104.0)	33.8%	58.2 (41.5)	7.8%
Smoking behaviour								
No	478.8 (107.9)	60.7%	135.2 (48.2)	17.2%	261.5 (107.5)	32.8%	64.9 (45.0)	8.6%
Yes	476.3 (104.6)	62.4%	136.4 (52.1)	17.9%	259.8 (106.0)	33.6%	64.0 (42.9)	8.7%
Drinking alcohol								
Never	461.7 (111.1)	58.2%	136.8 (49.6)	17.2%	250.7 (104.5)	31.3%	58.7 (40.8)	7.7%
1–7 glasses/week	475.3 (99.2)	60.9%	136.8 (47.9)	17.5%	256.1 (97.2)	32.6%	66.0 (44.6)	8.7%
> 7 glasses/week	496.1 (112.2)	63.9%	131.4 (49.6)	17.2%	276.2 (120.0)	35.0%	69.1 (48.1)	9.3%
Eating take-away food								
Never	503.1 (113.3)	60.8%	140.6 (52.9)	17.1%	292.3 (115.5)	35.0%	54.8 (42.9)	6.8%
Less than once a month	473.6 (104.5)	60.1%	140.9 (47.2)	17.9%	255.1 (103.6)	32.0%	62.5 (40.9)	8.2%
Once a month or more	465.6 (102.5)	61.8%	128.5 (47.0)	17.1%	246.1 (100.5)	32.4%	72.4 (46.4)	9.9%
Happiness								
Very happy/happy	478.2 (107.3)	61.1%	135.3 (48.5)	17.4%	260.3 (107.2)	32.9%	65.6 (44.4)	8.7%
Not happy	477.1 (104.7)	59.5%	138.3 (58.1)	17.4%	276.0 (109.2)	34.1%	50.6 (43.3)	6.5%

*BM* Body Mass Index

The numbers shown are mean minutes/day and the mean percentages of the total waking, non-occupational time/day

Table 3 presents the results of the logistic regression analyses of potential correlates of high levels of total (non-occupational) sedentary time, defined as ≥69% of the waking, non-occupational time/day (≥75th percentile of the proportion of waking non-occupational time). The multivariable analysis showed that women were less sedentary than men, that participants aged 35–64 years were less sedentary than younger participants (18–34 years old), that obese participants were more sedentary than participants with a normal weight status, and that full-time employed participants were more sedentary than participants who were not working.

**Table 3:** Logistic regression analyses of socio-demographic and health-related characteristics and high levels of total (non-occupational) sedentary time

	OR (95% CI) ≥69% total sedentary time	
	Univariate	Multivariable
Gender		
Male (ref)	1.00	1.00
Female	**0.20 (0.16–0.26)**	**0.21 (0.15–0.29)**
Age		
18–34 years old (ref)	1.00	1.00
35–49 years old	**0.42 (0.30–0.59)**	**0.31 (0.21–0.46)**
50–64 years old	0.74 (0.53–1.01)	**0.60 (0.40–0.90)**
≥ 65 years old	0.80 (0.57–1.12)	0.94 (0.55–1.62)
Educational level		
Low (ref)	1.00	1.00
Middle	1.22 (0.91–1.62)	1.03 (0.72–1.46)
High	**1.45 (1.09–1.93)**	1.18 (0.83–1.68)
Work status		
Not working (ref)	1.00	1.00
Part-time	**0.68 (0.49–0.94)**	1.33 (0.84–2.12)
Full-time	**1.90 (1.46–2.47)**	**1.71 (1.13–2.58)**
Self-reported health		
Very good/good (ref)	1.00	
Not good	1.02 (0.74–1.41)	
Mobility issues		
No issues (ref)	1.00	
Issues	0.97 (0.69–1.38)	
Weight status		
Normal (BMI 18.5–24.9; ref)	1.00	1.00
Overweight (BMI 25.0–29.9)	**1.36 (1.06–1.75)**	1.27 (0.95–1.69)
Obese (BMI ≥30.0)	1.48 (1.00–2.21)	**1.67 (1.06–2.64)**
Sleep time		
Sleeping 7–9 h/day (ref)	1.00	
Sleeping < 7 or > 9 h/day	1.20 (0.95–1.52)	
Smoking behaviour		
No (ref)	1.00	
Yes	1.25 (0.96–1.64)	
Drinking alcohol		
Never (ref)	1.00	1.00
1–7 glasses/week	**1.58 (1.15–2.17)**	1.29 (0.90–1.85)
> 7 glasses/week	**2.11 (1.50–2.95)**	1.12 (0.76–1.66)
Eating take-away food		
Never (ref)	1.00	1.00
Less than once a month	0.90 (0.65–1.23)	0.89 (0.61–1.29)
Once a month or more	**1.36 (1.02–1.80)**	1.26 (0.89–1.79)
Happiness		
Very happy/happy (ref)	1.00	
Not happy	0.85 (0.51–1.43)	

*p* < 0.05 in bold. *OR* Odds Ratio*, CI* Confidence Interval*, ref* reference category*, BMI* Body Mass

The cut-point for high levels of total (non-occupational) sedentary time (≥69% of the total waking, non-occupational time/day) is based on the 75th percentile

The results of the univariate and multivariable logistic regression analyses of potential correlates of high levels of household, leisure and transport sedentary time are shown in Table 4. Which characteristics were statistically significantly associated, as well as the direction of those associations, differed across the domains. The strongest associations showed that employed participants had higher levels of transport sedentary time than participants who were not working, and that women had statistically significant lower levels of leisure sedentary time than men.

**Table 4:** Logistic regression analyses of socio-demographic and health-related characteristics and high levels of sedentary time

	OR (95% CI) ≥21% household sedentary time		OR (95% CI) ≥40% leisure sedentary time		OR (95% CI) ≥12% transport sedentary time	
	Univariate	Multivariable	Univariate	Multivariable	Univariate	Multivariable
Gender						
Male (ref)	1.00	1.00	1.00	1.00	1.00	1.00
Female	**0.74 (0.59–0.93)**	0.90 (0.68–1.18)	**0.35 (0.28–0.45)**	**0.37 (0.28–0.50)**	**0.36 (0.29–0.46)**	**0.49 (0.36–0.68)**
Age						
18–34 years old (ref)	1.00	1.00	1.00	1.00	1.00	1.00
35–49 years old	**2.08 (1.46–2.97)**	**1.90 (1.33–2.72)**	**0.63 (0.44–0.91)**	**0.55 (0.37–0.83)**	1.04 (0.77–1.39)	0.93 (0.66–1.30)
50–64 years old	**1.79 (1.24–2.59)**	**1.55 (1.05–2.30)**	**1.60 (1.14–2.25)**	1.14 (0.76–1.70)	**0.68 (0.50–0.95)**	0.81 (0.55–1.18)
≥ 65 years old	**1.56 (1.05–2.32)**	1.58 (0.96–2.60)	**3.13 (2.20–4.44)**	**2.08 (1.26–3.43)**	**0.15 (0.09–0.25)**	**0.43 (0.22–0.85)**
Educational level						
Low (ref)	1.00	1.00	1.00	1.00	1.00	1.00
Middle	**0.64 (0.49–0.85)**	**0.60 (0.44–0.81)**	**0.65 (0.49–0.86)**	0.96 (0.68–1.35)	**2.23 (1.65–3.02)**	1.25 (0.87–1.77)
High	**0.70 (0.53–0.92)**	**0.62 (0.46–0.85)**	0.93 (0.71–1.21)	**1.46 (1.04–2.07)**	**2.39 (1.76–3.23)**	1.22 (0.86–1.75)
Work status						
Not working (ref)	1.00	1.00	1.00	1.00	1.00	1.00
Part-time	0.91 (0.67–1.24)	1.09 (0.75–1.59)	**0.28 (0.20–0.39)**	**0.64 (0.42–0.99)**	**3.08 (2.13–4.46)**	**2.25 (1.43–3.56)**
Full-time	**1.54 (1.18–2.01)**	**1.75 (1.21–2.53)**	**0.61 (0.48–0.79)**	0.80 (0.54–1.17)	**9.12 (6.52–12.76)**	**4.25 (2.76–6.55)**
Self-reported health						
Very good/good (ref)	1.00		1.00	1.00	1.00	1.00
Not good	0.96 (0.69–1.33)		**1.52 (1.12–2.05)**	1.18 (0.79–1.77)	**0.43 (0.29–0.64)**	0.66 (0.40–1.09)
Mobility issues						
No issues (ref)	1.00		1.00	1.00	1.00	1.00
Issues	1.05 (0.75–1.48)		**1.70 (1.24–2.32)**	0.98 (0.63–1.52)	**0.30 (0.19–0.49)**	0.77 (0.43–1.37)
Weight status						
Normal (BMI 18.5–24.9; ref)	1.00		1.00	1.00	1.00	
Overweight (BMI 25.0–29.9)	1.07 (0.83–1.37)		**1.59 (1.24–2.03)**	1.29 (0.97–1.71)	1.01 (0.79–1.29)	
Obese (BMI ≥30.0)	0.92 (0.60–1.40)		**2.05 (1.40–3.00)**	**1.87 (1.21–2.90)**	0.97 (0.64–1.46)	
Sleep time						
Sleeping 7–9 h/day (ref)	1.00		1.00	1.00	1.00	1.00
Sleeping < 7 or > 9 h/day	1.04 (0.82–1.32)		**1.36 (1.08–1.71)**	1.22 (0.93–1.59)	**0.64 (0.50–0.81)**	0.88 (0.66–1.16)
Smoking behaviour						
No (ref)	1.00		1.00	1.00	1.00	
Yes	1.09 (0.83–1.43)		**1.39 (1.07–1.80)**	**1.62 (1.19–2.20)**	0.95 (0.72–1.25)	
Drinking alcohol						
Never (ref)	1.00		1.00	1.00	1.00	1.00
1–7 glasses/week	1.04 (0.78–1.38)		1.18 (0.87–1.60)	1.10 (0.78–1.57)	**1.43 (1.06–1.94)**	1.10 (0.79–1.54)
> 7 glasses/week	0.89 (0.64–1.23)		**2.09 (1.52–2.87)**	1.29 (0.89–1.89)	**1.65 (1.19–2.28)**	1.17 (0.80–1.70)
Eating take-away food						
Never (ref)	1.00		1.00		1.00	1.00
Less than once a month	1.26 (0.93–1.70)		0.81 (0.60–1.09)		**1.66 (1.19–2.33)**	1.15 (0.78–1.69)
Once a month or more	0.97 (0.73–1.28)		0.87 (0.66–1.14)		**2.52 (1.85–3.42)**	**1.45 (1.01–2.06)**
Happiness						
Very happy/happy (ref)	1.00		1.00		1.00	
Not happy	1.02 (0.62–1.67)		1.28 (0.81–2.04)		0.70 (0.41–1.20)	

*p* < 0.05 in bold. *OR* Odds Ratio*, CI* Confidence Interval*, ref* reference category*, BMI* Body Mass Index

The cut-points for high levels of household sedentary time (≥21% of the total waking, non-occupational time/day), leisure sedentary time (≥40% of the total waking, non-occupational time/day), and transport sedentary time (≥12% of the total waking, non-occupational time/day) are based on the 75th percentiles

**Fig. 3:**
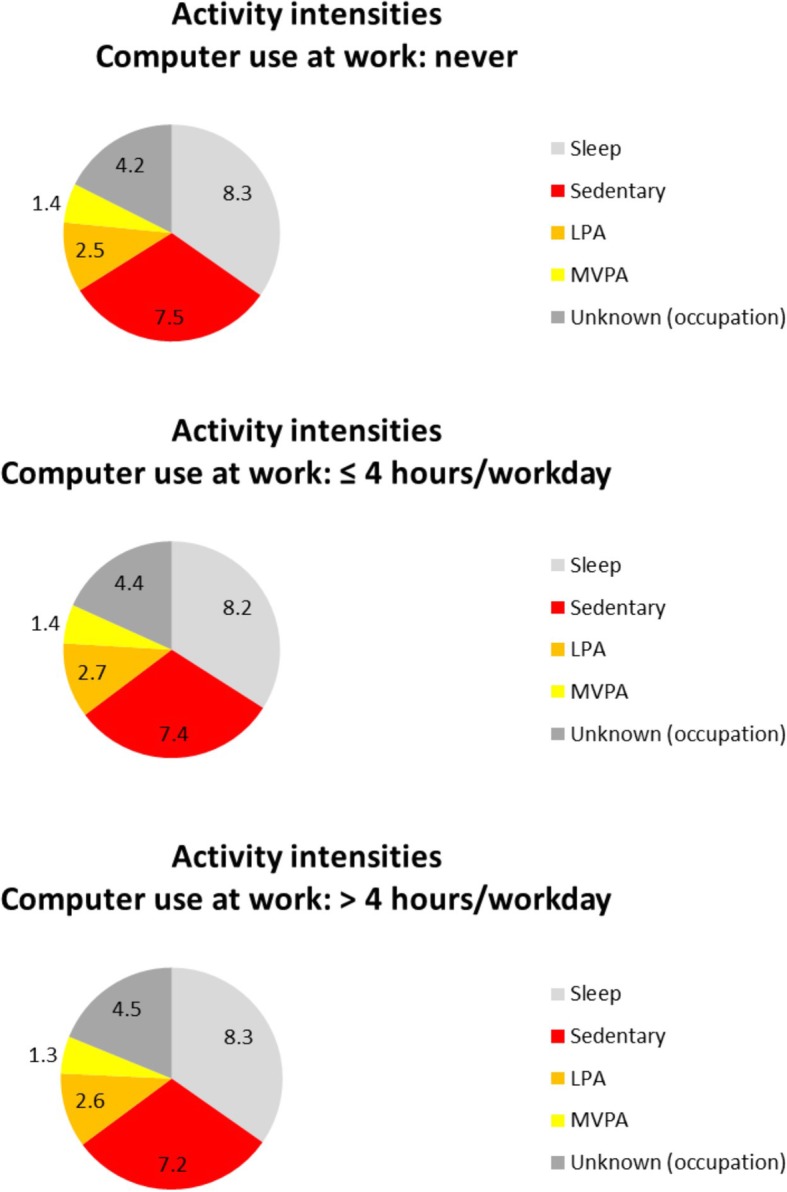
The average time (hours/day) spent in different activity intensities by full-time employed respondents. The results are shown separately for full-time employed respondents who never use a computer at work (above; *N* = 111), who use a computer ≤4 h/workday (middle; *N* = 165), and those who use a computer > 4 h/workday (below; *N* = 295). As occupational activities were not further specified, the intensity of these activities is unknown. *LPA = light intensity physical activity; MVPA = moderate to vigorous intensity physical activity*

Figure 3 shows the average time full-time employed participants spent in different activity intensities, stratified by computer use at work (never, ≤4 h/workday, > 4 h/workday). Across the three categories, the daily activity patterns are quite similar.

## Discussion

The current study investigated the time use of adults in the Netherlands according to the 2006 Dutch time use survey, as well as the potential correlates of high levels of sedentary time. On average, participants spent 8 h (60%) of their waking, non-occupational time on sedentary activities. Almost 90% of the time spent on leisure activities, and 70% of the time spent on transport activities, was sedentary. Men, participants aged 18–34 and 65+ years, full-time employed participants and obese participants were more likely to accumulate high levels of total (non-occupational) sedentary time. The correlates of household, leisure and transport sedentary time differed by domain.

Participants spent 8 h of their waking, non-occupational time/day on sedentary activities. As occupational activities could not be taken into account, and most occupations include at least some sedentary activities, this is likely an underestimation of total sedentary time – especially for people with sedentary occupations. Nevertheless, the current estimate is relatively high in comparison to previous studies using questionnaires. In a large European survey, for example, Dutch adults reported a median total sitting time of 6 h/day [6]. On the other hand, the current results are reasonably similar to a Dutch study using accelerometers to assess sedentary time, in which (relatively old) adult participants reported approximately 9 h of sedentary time per day [24]. Moreover, pooled results from multiple accelerometer studies in European countries also showed an average of 9 sedentary hours per day [25].

The results showed large differences in activity intensities across the different activity domains. Almost 90% of the leisure activities were sedentary. As people arguably have most autonomy in their leisure time, this might suggest a strong ‘natural’ inclination to be sedentary, but could also indicate that strategies aiming to reduce sedentary leisure time have great potential. Household activities were the largest source of LPA. This is notable, as there is increasing attention for the health benefits of LPA [26]. Even though active travel (cycling and walking) is common in the Netherlands, still 70% of time spent on transport activities was sedentary. It is possible, however, that relatively short active travel bouts, such as walking to the bus stop, were not detected in the current study due to the 10-min reporting interval of the time use diary. Nevertheless, time use data from 2006 from Australia, which has a stronger car-focussed infrastructure, shows a substantially higher proportion of sedentary transportation (83%) for Australian adults [20].

Gender, age, work status and weight status were associated with total (non-occupational) sedentary time, while the correlates of household, leisure and transport sedentary time differed by domain. The finding that men were more sedentary than women is in line with previous studies in a European context [6, 24, 25], although a systematic literature review reported less consistent results [8]. The domain-specific results showed that men spent a larger proportion of their waking, non-occupational time/day on leisure sedentary activities and transport sedentary activities than women, while there was no gender difference for sedentary household activities.

The current study showed higher sedentary levels for participants aged 18–34 and 65+ years. The current literature is mixed about the association between age and total sedentary time [8], which might be explained by the observed inconsistent findings across sedentary behaviour domains.

Full-time employed participants had higher levels of non-occupational sedentary time than people who were not employed. So, even though full-time employed participants had less non-occupational time, they spend a larger proportion of that time on sedentary activities. The domain-specific results showed that full-time employed participants spent a larger proportion of their time on household and transport sedentary activities. The higher proportion of sitting during household might reflect a lower participation in light intensity household tasks, while the higher proportion of sitting during transport might reflect the work commute. Furthermore, current findings showed that the daily activity patterns of full-time employed participants with different levels of occupational computer time were reasonably similar. As occupational computer time was used as a proxy indication for total occupational sedentary time, these results suggest that occupational sedentary time is additional to the daily non-occupational sedentary activities, and was not compensated for by being less sedentary in the non-work domains. People who sit more at work thus seem to be sitting more overall.

The finding that obese participants were more sedentary than participants with a normal weight is supported by most other studies [8, 25]. However, the current findings show that weight status is only associated with sedentary time during leisure. Leisure sedentary time is most frequently operationalised as television viewing, which has been associated with poorer eating habits and snacking behaviour in previous studies [27], though some research suggest that the association between sedentary behaviour and obesity is bi-directional [28].

Most studies have identified educational level as an important correlate of sedentary time [6, 8, 25], and have hypothesised that this is mostly due to differences in occupational sedentary time. That might explain why the current study did not find an association between educational level and total non-occupational sedentary time. The domain-specific results showed that participants with a higher educational level spent a smaller proportion of their waking, non-occupational time in household sedentary time, but a larger proportion in leisure sedentary time.

The lack of associations with several health-related characteristics such as self-reported health, sleep time, drinking alcohol, and happiness was also reported in a recent study looking at the correlates of (objectively measured) sedentary time in Dutch and Belgian adults [24].

Two recent studies investigated the correlates of leisure sedentary time in full-time employed Australian adults [29] and working adults in Singapore [30]. These studies also reported higher levels of leisure sedentary time for men [29, 30], obese participants [29], and current smokers [30]. This might indicate that men and people with an unhealthy lifestyle are more sedentary during leisure.

The finding that the correlates of high levels of household, leisure and transport sedentary time differed substantially by domain, demonstrates the importance of assessing the sedentary domains separately in research and surveillance. More insight is needed in order to appropriately address the different domains in interventions and policies aiming to decrease sedentary time.

### Strengths and limitations

The main strength of this study lies in the application of the Dutch time use survey data to assess the prevalence and correlates of sedentary behaviour. Even though time use surveys use the respondents’ description of their daily activities to classify their sedentary behaviours, without specifically assessing energy expenditure or posture, time use surveys have been shown to be a valid methodology to assess population levels of sedentary behaviour, with higher validity than more traditional surveillance methods such as questionnaires [11]. In addition, the detailed level of data enabled the study of different domains of sedentary behaviour, which provided new insights into the prevalence and correlates of sedentary time across these different domains.

One of the disadvantages of the current dataset is the fact that the data was already collected in 2006. Since then, there have been major technological developments, such as the widespread use of mobile devices, connectivity, and location-independent working, which have greatly influenced sedentary behaviours and especially screen time. As these changes are not reflected in the current data, the current manuscript did not focus on screen time behaviours. On the other hand, we purposefully used the 2006 Dutch time use survey data, as this is the most recent Dutch time use dataset that included important health-related variables such as BMI. This provided the unique opportunity to study these health-related characteristics in relation to sedentary behaviour.

Furthermore, a limitation of most time use surveys is that occupational activities are not reported in sufficient detail to assign activity intensities to them. Participants usually report they are ‘working’ without any indication of their specific activities. This means there is no complete 24-h data, which limits the interpretability of the results. In the current study, this was overcome by using the proportion of the waking, non-occupational time as an outcome. Moreover, (the influence of) occupational sedentary time was explored by looking at daily activity patterns of full-time employed participants with different levels of computer use at work. Nevertheless, time use survey designers are urged to think about ways to include more detailed information about the occupational activities of participants, for example by asking them to report the specific activities they perform at work.

## Conclusions

According to the 2006 Dutch time use survey, adults spent 8 h (60%) of their waking, non-occupational time/day on sedentary activities. As a portion of work time is also spent sedentary, total sedentary time will be substantial higher for the Dutch workforce and especially for people with a sedentary occupation. Almost 90% of the time spent on leisure activities was sedentary, which indicates that strategies aiming to reduce sedentary leisure time have great potential. Men, participants aged 18–34 and 65+ years, full-time employed participants and obese participants had higher levels of total (non-occupational) sedentary time. The correlates of household, leisure and transport sedentary time differed substantially by domain, which demonstrates the importance of targeting these domains differently in interventions and policies aimed at reducing sedentary time.

## Abbreviations

BMI: Body Mass Index
CI: Confidence Interval
HETUS: Harmonized European Time Use Survey
LPA: Light intensity Physical Activity
MET: Metabolic Equivalent
MVPA: Moderate-to-Vigorous Physical Activity
OR: Odds Ratio
SD: Standard Deviation
